# Animal Models of Nicotine Exposure: Relevance to Second-Hand Smoking, Electronic Cigarette Use, and Compulsive Smoking

**DOI:** 10.3389/fpsyt.2013.00041

**Published:** 2013-06-04

**Authors:** Ami Cohen, Olivier George

**Affiliations:** ^1^Committee on the Neurobiology of Addictive Disorders, The Scripps Research Institute, La Jolla, CA, USA

**Keywords:** addiction, tobacco, self-administration, vapor, dependence, escalation, abstinence, withdrawal

## Abstract

Much evidence indicates that individuals use tobacco primarily to experience the psychopharmacological properties of nicotine and that a large proportion of smokers eventually become dependent on nicotine. In humans, nicotine acutely produces positive reinforcing effects, including mild euphoria, whereas a nicotine abstinence syndrome with both somatic and affective components is observed after chronic nicotine exposure. Animal models of nicotine self-administration and chronic exposure to nicotine have been critical in unveiling the neurobiological substrates that mediate the acute reinforcing effects of nicotine and emergence of a withdrawal syndrome during abstinence. However, important aspects of the transition from nicotine abuse to nicotine dependence, such as the emergence of increased motivation and compulsive nicotine intake following repeated exposure to the drug, have only recently begun to be modeled in animals. Thus, the neurobiological mechanisms that are involved in these important aspects of nicotine addiction remain largely unknown. In this review, we describe the different animal models available to date and discuss recent advances in animal models of nicotine exposure and nicotine dependence. This review demonstrates that novel animal models of nicotine vapor exposure and escalation of nicotine intake provide a unique opportunity to investigate the neurobiological effects of second-hand nicotine exposure, electronic cigarette use, and the mechanisms that underlie the transition from nicotine use to compulsive nicotine intake.

## Introduction

Studies on the neurobiological substrates of tobacco addiction largely depend on the availability of suitable animal models. In this review, we first describe the features of tobacco smoking and nicotine abuse and dependence in humans. We then discuss the limits and advantages of the most used animal models of nicotine use and dependence and novel animal models of escalated nicotine intake and exposure to nicotine vapor. The last section discusses how these different animal models can be used to investigate the neurobiological mechanisms that mediate nicotine reinforcement and dependence.

## Features of Tobacco Smoking, Nicotine Abuse, and Dependence in Humans

Tobacco use is the leading cause of preventable disease and premature death, leading to 440,000 deaths annually in the United States alone (Fellows et al., 2002). According to a recent review (Giovino et al., 2012), 24% of the United States population older than 15 years of age are cigarette smokers, and 1.8% are smokeless tobacco users. Cigarette smoking appears to be more central to the epidemiology of nicotine addiction compared with smokeless tobacco abuse. However, chewing tobacco, dry snuff, and moist snuff are a concern in certain countries (Bhattacharyya, 2012; Giovino et al., 2012). The rapid growth of electronic cigarette use worldwide (Caponnetto et al., 2012) is also an important health concern that requires the development of novel animal models of exposure to nicotine vapor.

### Acute effects of smoking

The primary psychoactive ingredient responsible for tobacco use is nicotine (Cummings and Mahoney, 2006), although tobacco smoke also contains more than 4,000 additional chemicals, many of which have psychoactive properties or may act in concert with nicotine to contribute to smoking dependence (Clemens et al., 2009; Hoffman and Evans, 2013). Cigarettes typically contain 10–14 mg of nicotine (Kozlowski et al., 1998), of which 1–1.5 mg is absorbed systemically in the lungs through inhalation (Armitage et al., 1975; Benowitz and Jacob, 1984). Nicotine rapidly enters the pulmonary venous circulation, reaches the brain within 10–20 s, and readily diffuses into brain tissue where it binds to nicotine acetylcholine receptors (nAChRs; Benowitz et al., 1988). The rate of absorption of smokeless tobacco products, with the exception of electronic cigarettes, is considerably slower (30 min to reach maximum blood levels), accounting for a lower abuse potential for these products (Benowitz et al., 1988). Acutely, cigarette smoking is reported to induce positive reinforcing effects, including mild euphoria, heightened arousal, reduced appetite, and reduced stress, anxiety, and pain (Pomerleau et al., 1984; Pomerleau and Pomerleau, 1992; Stolerman and Jarvis, 1995). However, the specific role for nicotine in these reinforcing effects is still unclear because of the difficulties performing intravenous nicotine self-administration in humans. However, smokers who self-administer nicotine report an overall profile of rewarding sensations, including mild euphoria, increased comfort, “drug liking,” and reduced negative mood and pain sensation, accompanied by negative effects, such as tension and jitteriness (Henningfield and Goldberg, 1983; Perkins et al., 1994; Harvey et al., 2004; Sofuoglu et al., 2008; Rose et al., 2010). Thus, nicotine itself can serve as an effective reinforcer, at least among experienced smokers. However, the mixed subjective reports, early difficulties obtaining reliable intravenous nicotine self-administration in animals, and direct comparisons in animal models suggest that the reinforcing efficacy of nicotine is lower than other drugs of abuse (Risner and Goldberg, 1983; Manzardo et al., 2002; Le Foll and Goldberg, 2009). Non-nicotinic aspects of tobacco smoke, such as its other constituents (e.g., acetaldehyde, nornicotine, and harman) and sensory stimulation could substantially contribute to its abuse and addictive potential (Belluzzi et al., 2005; Rose, 2006; Rose et al., 2010; Kapelewski et al., 2011).

### Tobacco dependence

#### Patterns of smoking among dependent smokers

Dependent smokers maintain relatively stable nicotine blood levels during waking hours (Benowitz and Jacob, 1984), with plasma levels ranging between 20 and 50 ng/ml. To maintain these relatively constant nicotine levels, smokers efficiently regulate the rate and intensity of cigarette smoking (Ashton and Watson, 1970; Benowitz, 1986). For example, smokers will compensate for reduced nicotine content when smoking cigarettes with lower nicotine yield than their usual brand (Russell et al., 1980; Maron and Fortmann, 1987).

#### Nicotine withdrawal and the escalation of nicotine intake

Discontinuation of smoking, even for only several hours, leads to withdrawal symptoms that peak within 1 week and may persist for up to 6 months (Hughes et al., 1991; Hughes, 2007; Markou, 2008). Nicotine withdrawal includes both somatic symptoms, such as bradycardia, gastrointestinal disturbances, and, increased appetite, and affective symptoms, such as nicotine craving, heightened anxiety, hyperalgesia, depressed mood, and irritability (Pomerleau et al., 1984; Hughes et al., 1991; Zaniewska et al., 2009; Rose et al., 2010). Converging evidence shows that avoidance of the affective symptoms of nicotine withdrawal, rather than somatic symptoms, plays a central role in the maintenance of nicotine dependence (Koob et al., 1993). It has been hypothesized that during the transition to dependence, the motivation to take drugs is caused by a shift from the positive reinforcing properties of the drug to its ability to attenuate the negative effects of abstinence. Thus, the negative affective states associated with abstinence potentiate the incentive value of nicotine to promote the escalation of compulsive drug intake through negative reinforcement mechanisms (Solomon and Corbit, 1973; Koob and Le Moal, 2001; Koob, 2010).

#### Adolescence and the escalation of tobacco smoking

Tobacco smoking typically begins in adolescence, with 14% of 15-year-olds and 22% of 17-year-olds reporting cigarette smoking (Substance Abuse and Mental Health Services Administration, 2003). Prospective studies report that ∼30–50% of adolescents and young adults who had initiated non-daily smoking showed an escalation in daily smoking within 4–5 years (U.S. Department of Health and Human Services, 1994, 2012; Tucker et al., 2003). For example, one 4-year prospective study reports that 53% of sixth-graders who experimented with smoking experience dependence symptoms, and 40% experience escalation to daily smoking (Doubeni et al., 2010). Some adolescents and young adults who experiment with smoking will eventually quit or remain light smokers (one to five cigarettes/day) or intermittent smokers (“chippers”; Shiffman, 1989; Shiffman et al., 1994), a subpopulation that encompasses up to 25–33% of all smokers (Coggins et al., 2009).

Various psychosocial factors, such as peer smoking and parenting style, have been suggested to contribute to the escalated smoking behavior of certain adolescents (Robinson et al., 2003; Kim et al., 2009; Dal Cin et al., 2012). Interestingly, studies suggest that, contrary to the common perception, symptoms of nicotine dependence, most commonly craving for tobacco and withdrawal symptoms (Gervais et al., 2007; Doubeni et al., 2010; Zhan et al., 2012), can develop at very early stages of initial intermittent smoking, even with as few as two cigarettes per week (DiFranza et al., 2002). According to Zhan et al. (2012), 20% of adolescents who smoke fewer than 100 cigarettes in their lifetime report experiencing “smoking to relieve restlessness” and “irritability.” As expected, the early appearance of such symptoms of nicotine dependence predicts future escalation to daily chronic smoking (DiFranza et al., 2002, 2007; Dierker and Mermelstein, 2010; Doubeni et al., 2010). In contrast, people who engage in non-daily smoking without escalation (“chippers”) have very few or no symptoms of dependence, and their smoking experience is primarily associated with positive rather than negative reinforcement (Coggins et al., 2009). Thus, intermittent tobacco use associated with withdrawal symptoms can promote the escalation of smoking behavior, which in turn accelerates the appearance of additional symptoms of dependence (Doubeni et al., 2010).

The importance of nicotine withdrawal as a negative reinforcer in the escalation of smoking is also suggested by the calming effects of nicotine when given after even a short period of abstinence, a primary reason given by both adults and adolescents for smoking (Dozois et al., 1995; Parrott, 1995). Although nicotine has anxiolytic properties under certain conditions (Pomerleau et al., 1984; Perkins and Grobe, 1992; Juliano and Brandon, 2002), it has also been argued that the calming effects of nicotine in dependent smokers represent the reversal of the negative affect induced by nicotine deprivation (Parrott, 1995, 1998, 2003). Thus, escalation may be more common among individuals with difficulties regulating negative affect, who are prone to develop withdrawal symptoms, and who have high expectancy of the calming effects of smoking (Heinz et al., 2010).

### Second-hand smoke

One generally overlooked factor that may contribute to the escalation of tobacco abuse, particularly among adolescents, is second-hand smoking. In the United States, it has been estimated that up to 60% of children are exposed to second-hand smoke (U.S. Department of Health and Human Services, 2006). Nicotine from moderate second-hand smoke exposure results in an increase in plasma nicotine concentration of approximately 0.2 ng/ml and to substantial brain $\alpha_{4}\beta_{2}^{*}$ nAChR occupancy (19%) in both smokers and non-smokers compared with 0.87 ng/ml and 50% $\alpha_{4}\beta_{2}^{*}$ nAChR occupancy from actively smoking one cigarette (Brody et al., 2006, 2011). Although second-hand smoking is clearly linked to serious illnesses among non-smokers (U.S. Department of Health and Human Services, 2006), including asthma, heart disease, sudden infant death syndrome, and cancer, it is currently unclear whether second-hand smoke can also contribute to the initiation and escalation of smoking. It is well documented that adolescents exposed to smoking by family members and peers are more likely to initiate and escalate smoking behavior (Brook et al., 2009; Leonardi-Bee et al., 2011; Wang et al., 2011). However, various psychological, psychosocial, and genetic factors may mediate this effect (Ajzen and Fishbein, 1980; O’Byrne et al., 2002; Audrain-McGovern et al., 2007). Nevertheless, escalated smoking can be observed in adolescent smokers with cotinine plasma levels comparable to levels of second-hand smoking in non-smokers (DiFranza et al., 2007). Moreover, adults and children who are non-smokers report symptoms of nicotine withdrawal after exposure to high levels of second-hand smoke (Okoli et al., 2007; Bélanger et al., 2008). Finally, prospective studies suggest that high levels of nicotine intake from second-hand smoking during childhood predict smoking behavior in teenage years, even when accounting for various social and environmental factors (Becklake et al., 2005). However, the controlled experimental conditions that are required to test the causal role of second-hand smoking in the escalation of smoking can only be employed in animal models and will be discussed below.

### Electronic cigarettes

Electronic cigarettes deliver nicotine through the battery-powered vaporization of a nicotine/propylene-glycol solution. Electronic cigarettes (e-cigarettes) are thus generally less harmful than regular cigarettes because they deliver nicotine without the various toxic constituents of tobacco smoke (Cahn and Siegel, 2011; Etter and Bullen, 2011; O’Connor, 2012). According to a recent survey, 3.4% of the total population, including 11.4% of current smokers, 2.0% of former smokers, and 0.8% of never-smokers, use e-cigarettes (Pearson et al., 2012). Most smokers claim to use e-cigarettes for smoking cessation/reduction, and their use appears to enhance the motivation to quit (Etter and Bullen, 2011; Wagener et al., 2012). Indeed, two surveys reported that most smokers who used e-cigarettes decreased or completely quit smoking within 6 months (Polosa et al., 2011; Siegel et al., 2011). However, it is unclear the degree to which such reports coincide with the efficacy of e-cigarettes as nicotine delivery devices. Vansickel and Eissenberg (2013) report that experienced users who were allowed to use their own customized e-cigarettes reach blood nicotine concentrations similar to those obtained by regular cigarettes. However, other studies report that nicotine delivery greatly varies between brands but is generally lower than that of regular cigarettes, with certain brands delivering nicotine doses that are too low to be detected (Bullen et al., 2010; Vansickel et al., 2010; Goniewicz et al., 2013). These studies report that e-cigarette use reduces craving and partially alleviated withdrawal symptoms despite the low to moderate blood nicotine levels. The effect of e-cigarette use on the brain stress and reward systems and vulnerability to become dependent or relapse is unknown and needs to be addressed using novel animal models. Another key question that needs to be investigated is the possible role of e-cigarettes as a gateway product to other drugs of abuse (Etter, 2012).

## Animal Models of Nicotine Abuse and Dependence

### Non-contingent exposure to nicotine

**Figure 1:**
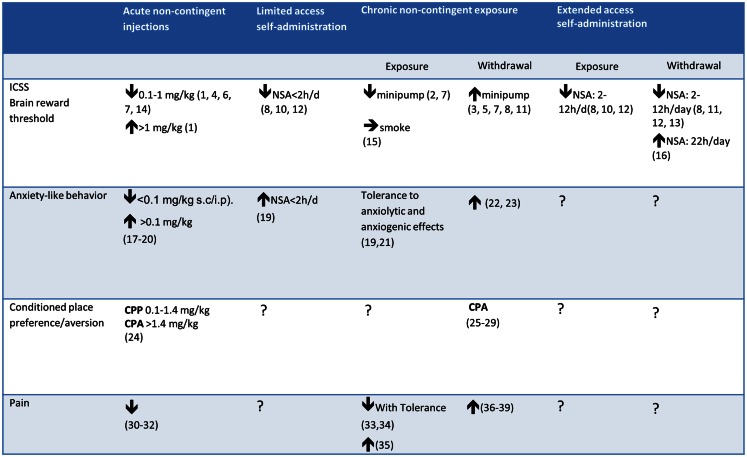
**Effects of acute/chronic non-contingent nicotine exposure, limited/extended access to nicotine self-administration (NSA), and withdrawal from chronic nicotine on measures of reward threshold (ICSS), anxiety-like behavior, and reward (CPP) or aversion (CPA)**. Note that the effect of withdrawal from chronic nicotine on the reward thresholds differed depending on the type of nicotine delivery. 1. Huston-Lyons and Kornetsky (1992), 2. Bozarth et al. (1998a), 3. Bozarth et al. (1998b), 4. Bespalov et al. (1999), 5. Watkins et al. (2000a), 6. Harrison et al. (2002), 7. Cryan et al. (2003), 8. Kenny and Markou (2005), 9. Kenny and Markou (2006), 10. Kenny et al. (2009), 11. Johnson et al. (2008), 12. Paterson et al. (2008), 13. Bruijnzeel et al. (2009), 14. Spiller et al. (2009), 15. Yamada et al. (2010) 16. Harris et al. (2011); 17. Brioni et al. (1993); 18. Irvine et al. (1999), 19. Irvine et al. (2001), 20. Tucci et al. (2003); 21. Biala and Budzynska (2006), 22. Stoker et al. (2008), 23. Cippitelli et al. (2011), 24. Le Foll and Goldberg (2005), 25. Miyata et al. (2011), 26. Suzuki et al. (1996), 27. Shram et al. (2008), 28. Grieder et al. (2012), 29. Grieder et al. (2010), 30. Damaj et al. (1994), 31. Sahley and Berntson (1979), 32. Craft and Milholland (1998), 33. Yang et al. (1992), 34. Galeote et al. (2006), 35. Lough et al. (2007), 36. Grabus et al. (2005), 37. Jackson et al. (2008), 38. Schmidt et al. (2001), 39. Yang et al. (1992).

Most research on the behavioral and biological effects of nicotine involved experimenter-administered nicotine, given by subcutaneous (s.c.) or intraperitoneal (i.p.) injections (see Figure 1). Non-contingent nicotine injections were instrumental in identifying the effects of acute and chronic exposure to nicotine on a wide variety of phenomena, including locomotor activity (Clarke and Kumar, 1983), anxiety-like behavior (Irvine et al., 1999; Cheeta et al., 2001), feeding behavior (Clarke and Kumar, 1984), pain (Sahley and Berntson, 1979), the development of tolerance to such effects (Collins et al., 1988), and the brain systems involved (Rosecrans and Meltzer, 1981; Clarke et al., 1988; Niijima et al., 2001).

#### Conditioned place preference

In this model of drug reward, animals are tested for the development of conditioned preferences for distinct drug-paired environments (Carr et al., 1989). Achieving nicotine-induced conditioned place preference (CPP) in rodents has proven to be challenging compared with other drugs of abuse, and findings have been inconsistent. Nicotine-induced CPP is observed in some studies (Fudala et al., 1985; Horan et al., 1997; Ashby et al., 2002; Le Foll and Goldberg, 2005) but not in others (Clarke and Fibiger, 1987; Acquas et al., 1989; Jorenby et al., 1990; Parker, 1992). Nicotine can also induce conditioned place aversion (CPA; Horan et al., 1997; Laviolette and van der Kooy, 2003). The ability to achieve nicotine-induced CPP is facilitated by the use of a “biased” place preference procedure (i.e., pairing the drug with the initially non-preferred compartment of the CPP apparatus; Le Foll and Goldberg, 2005). The reasons for the difficulty obtaining CPP are unclear and may be related to the weak rewarding properties of nicotine and very narrow dose-response curve.

#### Dependence induction

Termination of repeated nicotine injections in rodents results in behavioral and physiological states consistent with drug withdrawal (see review by Malin, 2001), such as heightened stress responses (Benwell and Balfour, 1979), the disruption of appetitive operant responding (Ford and Balster, 1976; Carroll et al., 1989), and weight gain (Grunberg et al., 1986; Levin et al., 1987). The induction of nicotine dependence by subcutaneous nicotine delivery via osmotic minipumps has gained popularity since its first introduction by Malin et al. (1992). In this method, dependence is induced by ≥6 days of continuous subcutaneous nicotine infusion (commonly ≥3.16 mg/kg free base/day in rats and ≥12 mg/kg/day in mice). Withdrawal is subsequently induced by terminating the infusion (peaking within 18–22 h; Malin et al., 1992) or precipitated by injecting nAChR antagonists, such as mecamylamine (Malin et al., 1992; Isola et al., 1999; Damaj, 2000; Malin, 2001). The symptoms of withdrawal are commonly divided into “somatic” signs that resemble opiate withdrawal (e.g., teeth-chattering, chewing, writhing, tremors, and body shakes; Malin et al., 1992). Although a well-accepted marker for nicotine dependence, these somatic withdrawal signs do not appear to be similar to those in humans or strongly predict drug use or relapse compared with affective symptoms (Koob and Le Moal, 2001; Hughes, 2007). Affective symptoms can be measured using CPA to nicotine withdrawal (Shram et al., 2008; Jackson et al., 2009), anxiety-like behavior (Wilmouth and Spear, 2006), and increased reward thresholds in the intracranial self-stimulation (ICSS) paradigm. The increased reward thresholds are interpreted as reflecting a state of dysphoria or reduced ability to experience reward (Watkins et al., 2000a; Vlachou et al., 2011). Hyperalgesia, a withdrawal symptom that may be considered partly somatic and partly affective, is also observed in rodents following spontaneous or mecamylamine-induced withdrawal from chronic non-contingent nicotine delivery (Schmidt et al., 2001; Damaj et al., 2003; Jackson et al., 2009, 2010). Hyperalgesia in such studies is operationally defined as increased sensitivity to nociceptive stimuli, usually in the form of tail-flick or hot-plate tests of latency to respond to noxious thermal stimuli.

Non-contingent exposure to nicotine is a simple and efficient way to induce nicotine dependence in animals and led to a great deal of findings regarding the possible neurobiological mechanisms of reward, dependence, and withdrawal (Malin, 2001; Malin and Goyarzu, 2009). However, the validity of this approach is limited when one wants to specifically investigate the neurobiological mechanisms that underlie the transition from occasional to compulsive use. Most importantly, contingent drug exposure (i.e., cigarette smoking and nicotine self-administration) and non-contingent exposure have very different psychological and physiological effects and recruit different brain systems (Dworkin et al., 1995; Markou et al., 1999). Nicotine absorption through subcutaneous or intraperitoneal administration is much slower than that achieved through inhalation, and the speed of administration has been shown to critically influence the reinforcing effects of drugs of abuse (Liu et al., 2005; Sorge and Clarke, 2009; but see Crombag et al., 2008). While minipumps deliver nicotine 24 h per day at a constant rate, humans smoke nicotine intermittently and not during sleep. Finally, the daily amount of nicotine typically delivered by minipumps (3.16 mg/kg) is similar to an average adult who smokes five packs of cigarettes, an amount consumed only by exceptionally heavy smokers (Armitage et al., 1975; Benowitz and Jacob, 1984). However, when differences between the metabolic rate of rats (nicotine half life = 45 min; Adir et al., 1976; Plowchalk et al., 1992) and humans (half life = 2 h) are taken into account, the actual disparity between the amounts absorbed is minimized (Malin, 2001), although comparisons remain difficult.

### Nicotine self-administration

The self-administration method assesses an animal’s propensity to self-administer a drug delivered (usually intravenously) contingently upon the emission of an operant response, usually a lever-press or nosepoke (Meisch and Lemaire, 1993). Since the early 1980s, an increasing number of laboratories have reported reliable rates of operant responding in nicotine self-administration studies with rodents (Corrigall and Coen, 1989; Donny et al., 1995; Watkins et al., 1999; Corrigall et al., 2000), but compared with other drugs of abuse, stable rates of nicotine self-administration remains difficult to establish and require careful control of a relatively high number of experimental parameters, such as the drug infusion duration, prior food training, restricted diets, and the need for cued infusions of nicotine (Henningfield and Goldberg, 1983; Collins et al., 1990; Stolerman and Jarvis, 1995; Le Foll and Goldberg, 2005; Chaudhri et al., 2006). At least some of the described difficulties obtaining nicotine self-administration may be related to the aversive properties of the drug (Benowitz, 1990). The difference between the rewarding and aversive doses of nicotine appears to be relatively small. Specifically, rats will intravenously self-administer nicotine at doses of 0.01–0.06 mg/kg (e.g., Corrigall and Coen, 1989; Donny et al., 1995; Shoaib et al., 1997), while an intravenous nicotine dose of 0.1 mg/kg has been reported to cause seizures (Hanson et al., 1979; Corrigall and Coen, 1989). Thus, when the behavioral criteria for demonstrating nicotine’s reinforcing properties require that animals repeatedly self-administer the drug, the likelihood of an accumulating blood nicotine concentration that is no longer within the reinforcing dose range is greatly elevated (see Rose and Corrigall, 1997).

### Escalation of nicotine self-administration

Rats allowed 1–3 h/day access to nicotine self-administration maintain stable and relatively low intake for weeks, exhibit very limited, if any, spontaneous withdrawal symptoms, and do not show increased motivation for nicotine after abstinence (Paterson and Markou, 2004; George et al., 2007; Cohen et al., 2012). The model of limited access to drug self-administration is highly relevant to the positive-reinforcement processes that account for the initiation and maintenance of occasional/recreational drug users but not for the transition to drug dependence, which is characterized in humans by escalated drug intake (Koob et al., 2004), robust somatic and affective withdrawal symptoms, and most importantly increased motivation for nicotine after protracted abstinence (Perkins et al., 2009). In contrast, rats exposed to extended (6–23 h) daily opiate, cocaine, or methamphetamine self-administration sessions show escalation in drug intake (Ahmed and Koob, 1998, 1999; Ahmed et al., 2000; Ben-Shahar et al., 2004; Greenwell et al., 2009) that is characterized by an upward shift in the dose-effect function that could not be simply explained as the result of a change in the sensitivity to the drug (i.e., pharmacological tolerance or sensitization; Koob and Le Moal, 1997; Ahmed and Koob, 1998). It has been hypothesized that the escalation of drug intake reflects an allostatic increase in the hedonic set point as a result of downregulation of brain reward systems and recruitment of brain stress systems (Ahmed and Koob, 1998; Koob and Kreek, 2007). In line with this hypothesis, the escalation of opiate and cocaine intake is correlated with a progressive elevation in baseline reward thresholds (Ahmed et al., 2002; Kenny et al., 2006). Further supporting the validity of the escalation model for human addiction, the escalation of cocaine self-administration has also been shown to be accompanied by increased susceptibility to reinstatement (Mantsch et al., 2004; Wakabayashi et al., 2010) and increased stress reactivity (Aujla et al., 2008). However, the escalation of nicotine intake is not observed when rats are allowed daily extended access (6–24 h/day; 20–40 days) to nicotine (Cox et al., 1984; Valentine et al., 1997; DeNoble and Mele, 2006; Kenny and Markou, 2006; O’Dell et al., 2007), despite exhibiting levels of nicotine intake similar to human smokers (rats: 0.2–1.5 mg/kg/day; humans: 0.14–1.14 mg/kg/day; Benowitz and Jacob, 1984), and physical dependence as measured by spontaneous and mecamylamine-precipitated somatic signs (Paterson and Markou, 2004; O’Dell et al., 2007). Moreover, in contrast to the increased reward thresholds observed after extended access to cocaine, heroin, and methamphetamine, repeated exposure to nicotine self-administration (1–12 h/day for 20 days) has been shown to induce a long-lasting decrease in reward thresholds (Kenny and Markou, 2006), a result opposite to that observed after chronic exposure to osmotic minipumps (Epping-Jordan et al., 1998; Watkins et al., 2000a; see Figure 1). These results suggest either that nicotine dependence differs from dependence on the other drugs of abuse or that modeling the transition to escalation of compulsive nicotine intake requires revision of the existing model.

As discussed above, nicotine dependence commonly develops as adolescents and young adults who smoke intermittently escalate their drug intake. It has been repeatedly shown that intermittent access to alcohol leads to higher levels of alcohol intake than continuous access, suggesting that neurobiological changes that underlie dependence may be more readily triggered by repeated cycles of withdrawal followed by increased intake (Sinclair and Senter, 1967; O’Dell et al., 2004; Lopez and Becker, 2005; Becker and Baros, 2006). Thus, a model of dependence-induced excessive nicotine intake was developed in our laboratory, in which rats are allowed to self-administer nicotine 4 days per week for either 23 h/day (extended access) or 1 h/day (limited access), followed by 2–3 days of abstinence. Rats with extended access exhibit a pronounced increase in nicotine intake in the first post-abstinence session, with a gradual return to baseline intake levels within the remaining three daily sessions (George et al., 2007; O’Dell and Koob, 2007). This nicotine deprivation effect does not occur in rats with limited access to nicotine, suggesting that the extended-access model has better validity for studying the increased motivation for nicotine during abstinence. Moreover, 1–12 h/day of access to nicotine self-administration results in either decrease or no change in brain reward threshold during abstinence (Kenny and Markou, 2006; Patterson et al., 2008), while extending the access to 22 h/day produces an increase in brain reward threshold during the first 3 days of abstinence (measured during extinction of nicotine self-administration, Harris et al., 2011). This result is in accordance with the increase in brain reward threshold observed during withdrawal (Epping-Jordan et al., 1998) and conditioned withdrawal (Kenny and Markou, 2005) after chronic exposure to nicotine minipump, and with the increase dysphoria, depressed mood, anxiety, and frustration reported in humans during abstinence (Hughes et al., 1991).

Based on these results, we developed a novel animal model of the escalation of nicotine intake, in which rats have extended (21 h/day) but intermittent (every 24–48 h) access to nicotine self-administration (0.03 mg/kg). Escalation occur only when the rats are allowed extended but not limited access (Cohen et al., 2012), and is associated with increased motivation to take nicotine on a progressive-ratio schedule of reinforcement and with a more intense somatic signs following precipitated withdrawal. In line with the hypothesis that tobacco smoking is more reinforcing/addictive than pure nicotine because of non-nicotine compounds, such as monoamine oxidase inhibitors (MAOIs; Berlin and Anthenelli, 2001; Fowler et al., 2003; Guillem et al., 2005, 2006), the escalation is dramatically increased when rats are pretreated with the MAOI phenelzine (2 mg/kg, i.p.,) prior to each extended-access self-administration session.

As stated above, limited access (1–12 h/day) to nicotine self-administration does not produce escalation of nicotine intake, however, a recent report showed that rats with limited access to nicotine self-administration (2 h/day) escalate their nicotine intake if they are given access to nicotine 8–12 h into withdrawal from exposure to nicotine vapor (Gilpin et al., 2013). Considering that this exposure to nicotine vapor was sufficient to produce robust withdrawal symptoms (Gilpin et al., 2013), it suggests that emergence of a negative withdrawal syndrome is required for the development of escalation of nicotine intake (George et al., 2007; Gilpin et al., 2013), and suggest that exposure to nicotine vapor either passively (second-hand smoking) or actively (electronic cigarette) may have profound consequences on the acquisition and relapse of smoking behavior.

### Effects of nicotine exposure and withdrawal in adolescence

Converging lines of evidence suggest that adolescence is a vulnerable period in the development of tobacco addiction (O’Dell, 2009). Specifically, compared to adult, adolescent rats show increased sensitivity to the rewarding effects of nicotine as measured with both self-administration (Levin et al., 2003; Chen et al., 2007) and the CPP procedures (Belluzzi et al., 2004; Shram et al., 2006; Torres et al., 2008). On the other hand, adolescent rats demonstrate lower aversive responses to high nicotine doses measured with CPA and conditioned taste aversion (Shram et al., 2006; Torres et al., 2008). Interestingly, adolescent rats may be more sensitive also to the contribution of non-nicotinic tobacco smoke ingredients of tobacco as acetaldehyde, a major component of tobacco smoke, appears to more readily enhance nicotine self-administration in adolescent but not adult rats (Belluzzi et al., 2005).

In addition to the increased rewarding effects and reduced aversive effect of nicotine in adolescents, studies using models of withdrawal from chronic passive nicotine delivery suggest that adolescent rats have a more benign nicotine withdrawal syndrome, as reflected by lower levels of somatic signs (O’Dell et al., 2004; Shram et al., 2008), withdrawal thresholds (O’Dell et al., 2006), CPA (O’Dell et al., 2007), and anxiety-like behavior in the elevated plus maze (Wilmouth and Spear, 2006).

Importantly, the human data on adolescence as a critical period in the establishment of smoking behavior in adulthood is supported by the finding that exposure to nicotine during adolescence is associated with enhanced rewarding effects of nicotine. For example, adult rats that initiated nicotine self-administration during adolescence, show higher levels of nicotine intake than rats that initiated nicotine self-administration during adulthood (Adriani et al., 2003) and rats that received nicotine during adolescence show in adulthood greater nicotine-induced place preference (Adriani et al., 2006) and increased anxiety induced withdrawal (Slawecki et al., 2003). However, the transition from nicotine use to nicotine addiction (i.e., escalation) has not yet been examined in adolescent rats.

### Exposure to cigarette smoke and nicotine vapor

Animal models that utilize inhalation as the route of administering cigarette smoke or nicotine have exceptional face validity because they best simulate the unique pharmacokinetic characteristics (i.e., rate of absorption and brain delivery) that are associated with smoking, which may have implications for its addictive properties (Benowitz, 1990). Moreover, the stimulation of the respiratory tract by tobacco smoke (e.g., by local nicotinic receptors; Ginzel and Eldred, 1977) may play a role in nicotine dependence (Rose and Corrigall, 1997). Another advantage of inhalation-based models is that they are non-invasive and much less labor-intensive than those that involve osmotic minipumps. Although current inhalation technology allows only for non-contingent passive exposure and not for self-administration, it is particularly suitable for the study of the detrimental effects of second hand smoke and their contribution to addiction in particular.

Automated smoke machines that deliver cigarette smoke to animals in exposure chambers have been used extensively to study the toxic effects of mainstream and sidestream (“second hand”) tobacco smoke (Hecht, 2005; Farkas et al., 2006; Coggins, 2007). Particularly, chronic exposure to sidestream smoke simulating environmental tobacco smoke has been recently shown to induce behavioral and neurobiological changes in laboratory animals. In primates, prenatal and postnatal environmental smoke exposure induces neuronal damage to the cortex and midbrain (Slotkin et al., 2006) and impaired memory (Golub et al., 2007). In rats, chronic exposure during postnatal days 8–23 leads to perturbed mitochondrial processes in the cerebellum that is associated with a heightened locomotor response in a novel environment (Fuller et al., 2012). Similar chronic exposure during adulthood results in biochemical changes in several brain regions (hippocampus, cerebellum, frontal cortex) indicative of enhanced inflammatory processes and cell death (Fuller et al., 2010) as well as in learning and memory impairments (Jaques et al., 2012).

Repeated exposure to mainstream cigarette smoke (modeling exposure of active smokers) induces effects similar to those of nicotine injections, including nAChR-dependent analgesia in rats, with the development of tolerance following repeated exposures (Anderson et al., 2004; Simons et al., 2005), sensitization to the effects of nicotine on locomotion (Suemaru et al., 1992; Bruijnzeel et al., 2009), and dependence as indicated by mecamylamine-precipitated somatic withdrawal signs and elevated reward thresholds (Small et al., 2010; Yamada et al., 2010). Small et al. (2010) reports that despite induction of a dependent state, nicotine self-administration is decreased 24 h after the termination of 28 consecutive tobacco smoke exposure sessions (4 h/day) and returns to control levels 5 days later. However, these results need to be interpreted with caution because the levels of nicotine and carbon monoxide to which the rats were exposed were very high in most of these studies. For example, average plasma nicotine levels in dependent smokers are 10–50 ng/ml (Russell et al., 1980; Benowitz and Jacob, 1984; Henningfield and Keenan, 1993), and average blood carboxyhemoglobin (COHgb) saturation, resulting from carbon monoxide exposure, is 4–10% (Benowitz et al., 1982; Turner et al., 1986; Law et al., 1997). Plasma nicotine concentrations in the cigarette smoke exposure studies described above ranged from 38.5 (Bruijnzeel et al., 2009) to 95.4–188 ng/ml (Anderson et al., 2004; Small et al., 2010; Yamada et al., 2010). Although COHgb levels were not reported, carbon monoxide levels in the chambers [150–402 parts per million (PPM)] were 40–400% higher than the level needed to induce COHgb saturation of 10.5% (Harris et al., 2010). These are especially high compared with the values in non-smokers exposed to second-hand smoke (5.9 ng/ml of serum nicotine; Pacifici et al., 1995) and carbon monoxide levels of 5–20 PPM (Office of Technology Assessment, 1986), leading to COHgb levels of 4.43% (Yee et al., 2010). In addition to nicotine, tobacco smoke contains at least 4,000 additional substances, many of which are toxic or psychoactive, further complicating data interpretation. For example, rats exposed to high levels of carbon monoxide and other toxins may develop adverse effects that will hinder their motivation to take nicotine. Alternatively, some components of tobacco smoke may negate certain effects of nicotine. This could explain the finding that although daily nicotine (0.125 mg/kg, s.c.) reverses the elevated reward thresholds induced by withdrawal from chronic nicotine, cigarette smoke exposure that induces the same serum nicotine levels (25–55 ng/ml) did not (Harris et al., 2010). Thus, although cigarette smoke exposure uniquely allows the determination of the net effect of tobacco smoke, isolating the specific effects of different components of tobacco smoke is difficult.

The recently developed model of nicotine vapor (George et al., 2010; Gilpin et al., 2013) addresses this shortcoming. The vaporization of nicotine is achieved without the use of heat by constantly bubbling nicotine with air and allowing for the reliable induction of air-nicotine concentrations that induce blood nicotine levels comparable to those of different tobacco exposure levels (heavy smokers, moderate smokers, and second-hand smoking). Intermittent exposure to nicotine vapor (0.2 mg/m^3^ for 8 h/day for 7 days) produces a concentration of nicotine in the blood of 22 ng/m, which is in the range of moderate smokers, and induces significant somatic withdrawal signs precipitated by mecamylamine (George et al., 2010). The concentration of nicotine in vapor chamber air can be adjusted to produce blood nicotine levels that are relevant to heavy, regular, or second-hand smoking and e-cigarette use. Moreover, as stated above, rats exposed to nicotine vapor (7.5 mg/m^3^ over a 12-h period) to the point of dependence produce an escalation of nicotine self-administration relative to both their own baseline (200% increase) and non-dependent controls.

Thus, models based on the inhalation of tobacco smoke or pure nicotine have the potential to reliably detect the biological mechanisms that are unique to the consumption of tobacco via smoking and determine the possible contribution of constituents in second-hand smoke, particularly nicotine, in the transition to nicotine dependence, reflected by the escalation of nicotine intake. Future studies will need to address this issue using relatively low levels of nicotine/smoke exposure and examine the effects of exposure to a combination of nicotine and certain other selected constituents of tobacco smoke (e.g., acetaldehyde and harman) on different aspects of tobacco dependence. Finally, nicotine vapor is the only model available to date that can be used to investigate the neurobiological effects of nicotine delivery by e-cigarettes on the vulnerability to develop nicotine dependence and relapse.

## Neurobiological Mechanisms of Nicotine Addiction

The different animal models of nicotine abuse and dependence have been widely used to unveil the neurobiological mechanisms that mediate the acute and chronic effects of nicotine. Models of the acute reinforcing effects of nicotine were established more than two decades ago, and the biological processes involved are well-characterized. In contrast, the neurobiological mechanisms that mediate the increased motivation for nicotine associated with drug dependence are poorly known.

### Acute effects of nicotine

#### Nicotine acetylcholine receptors

Nicotine acetylcholine receptors are distributed throughout the central nervous system (Paterson and Nordberg, 2000), and their activation increases the release of various neurotransmitters (Wilkie et al., 1993; McGehee et al., 1995; Clarke and Reuben, 1996; Pontieri et al., 1996; Yang et al., 1996). The acute reinforcing and rewarding effects of nicotine are mediated by the activation of nAChRs, which are composed of five subunits that can either be homomeric or heteromeric (Millar and Gotti, 2009). Twelve different neuronal nAChR subunits (α2–α10 and β2–β4) have been identified (Dani and Bertrand, 2007). Inactivation of α7-, α4-, α6-, and β2-containing nAChRs by pharmacological or genetic manipulations decrease nicotine self-administration in rodents (Picciotto et al., 1988; Dwoskin et al., 1999; Markou and Paterson, 2001). These subunits likely mediate the acute reinforcing effects of nicotine. In contrast, α5 knockout mice show increased nicotine self-administration at a high unit dose, suggesting the involvement of this subunit in mediating the aversive effects of high nicotine doses (Fowler et al., 2011).

#### Mesocorticolimbic system: dopamine

The acute reinforcing effects of nicotine and other drugs of abuse are in part mediated by activation of the mesocorticolimbic dopamine system (Koob and Le Moal, 2008). The mesocorticolimbic dopamine system includes dopaminergic neurons that originate in the ventral tegmental area (VTA) and project to the nucleus accumbens (NAc), hippocampus, amygdala, and prefrontal cortex (PFC). Indeed, nicotine exposure increases dopamine release in mesolimbic terminal fields (Di Chiara, 2000). Rats will self-administer nicotine directly into the VTA (Ikemoto et al., 2006), and intra-VTA infusion of a nicotine antagonist decreases nicotine self-administration (Corrigall et al., 1994). In addition, disruption of dopamine transmission either systemically or in the VTA attenuates nicotine self-administration (Corrigall and Coen, 1991) and prevents the reduction of brain reward thresholds induced by nicotine (Huston-Lyons et al., 1993). In the place preference procedure, dopamine antagonists block nicotine-induced CPP (Acquas et al., 1989), but in a study by Laviolette and van der Kooy (2003), nicotine infusion into the VTA dose-dependently induced CPA at low dose and CPP at high doses, and systemic infusion of a dopamine antagonist potentiated the rewarding effects of mid-range nicotine doses and switched the motivational effects of a low concentration from aversive to rewarding. These results appear to be contradictory to those obtained with the self-administration model (Ikemoto et al., 2006) and may suggest different roles for dopamine in mediating specific functions of reward and reinforcement that may be dose-dependent.

#### Glutamate, GABA, and acetylcholine

Nicotine increases dopamine neurotransmission in the mesocorticolimbic system by activating nAChRs, particularly α4β2, on dopaminergic neurons in the VTA (Nisell et al., 1994; Mansvelder and McGehee, 2003) and nAChRs, particularly α7-containing glutamatergic neurons that originate in the VTA, NAc, amygdala, hippocampus, and PFC (Fu et al., 2000; Mansvelder and McGehee, 2003) and project to dopaminergic neurons in the VTA (Grillner and Svensson, 2000). Consequently, antagonists of various glutamate receptors, including NMDA, AMPA, and mGluR5, decrease nicotine self-administration, whether delivered systemically or into the VTA (Kenny et al., 2003, 2009; Patterson et al., 2003; Liechti and Markou, 2008), and NMDA and AMPA receptor antagonists block nicotine-induced dopamine release in the NAc (Kosowski et al., 2004). Moreover, lesions of glutamatergic inputs from the pedunculopontine tegmental nucleus (PPT) to VTA inhibit nicotine self-administration and CPP (Lança et al., 2000; Laviolette et al., 2002; Picciotto and Corrigall, 2002). The PPT also contains cholinergic neurons that are activated by nicotine and project to dopaminergic neurons in the VTA. Indeed, delivery of an antagonist of non-α7 nAChRs to the PPT or lesions of cholinergic neurons in the PPT reduced nicotine self-administration (Lança et al., 2000; Corrigall et al., 2001, 2002; Alderson et al., 2006). Finally, intra-VTA GABAergic neurons are activated by nicotine and inhibit dopamine neurons. However nAChR on GABAergic neurons desensitize faster than nAChRs on dopamine neurons, leading to a facilitation of dopamine neuron firing (Laviolette and van der Kooy, 2004). Accordingly, enhanced activation of GABA_B_ receptors inhibits nicotine self-administration and CPP in rats (Patterson et al., 2004, 2008; Le Foll et al., 2008).

#### Endogenous opioids

The endogenous opioid system may also play an important role in the rewarding and reinforcing effects of nicotine (for review, see Berrendero et al., 2010). Briefly, endogenous opioid systems include three main receptors, μ (MOR), δ (DOR), and κ (KOR; Kieffer and Evans, 2009). Of the opioid peptides in the brain, β-endorphin binds with a higher affinity to MORs than DORs or KORs, and it is a main endogenous ligand for MORs. Dynorphins are the main endogenous ligands for KORs (Roth-Deri et al., 2008). Nicotine enhances the release of endogenous opioid peptides and modifies the expression of their receptors. For example, acute nicotine induces increases in met-enkephalin, dynorphin, and prodynorphin mRNA in the striatum of mice after acute nicotine injection (Dhatt et al., 1995; Isola et al., 2009). Nicotine-induced dopamine increase in the NAc can be blocked by the administration of MOR antagonists or KOR agonists (Maisonneuve and Glick, 1999). However, although systemic inhibition of β-endorphin-MORs by pharmacological or genetic manipulations generally reduces the rewarding effects of nicotine in animal models (Berrendero et al., 2002; Göktalay et al., 2006; Trigo et al., 2009), the blockade of opioid receptors in the VTA and NAc does not interfere with nicotine self-administration in rats (Corrigall and Coen, 1991; Corrigall et al., 2000). Interestingly, prodynorphin knockout mice show enhanced acquisition of nicotine self-administration (Galeote et al., 2009), suggesting that the prodynorphin-KOR system may mediate the aversive effects of nicotine, particularly at high doses, as was demonstrated with other drugs of abuse (Mendizábal et al., 2006; Shippenberg et al., 2007).

#### Serotonergic system

Serotonin [5-hydroxytryptamine (5-HT)] neurons in the median and dorsal raphe nuclei provide the majority of 5-HT innervation to the forebrain and are associated with appetitive behavior and affect regulation (Steinbusch, 1984). Their involvement in nicotine reinforcement is suggested by nicotine-induced increases in dorsal raphe neuron firing and 5-HT release (Ribeiro et al., 1993; Li et al., 1998; Mihailescu et al., 1998, 2002; Martinez-Gonzalez et al., 2002). Agonists of 5-HT_2C_ receptors reduce nicotine self-administration (Grottick et al., 2001) but not nicotine-induced CPP (Hayes et al., 2009).

#### Endocannabinoids

Endocannabinoid systems may also be involved in the rewarding and reinforcing effects of nicotine. CB_1_ receptor antagonists decrease nicotine self-administration and CPP in rodents (Cohen et al., 2002; Le Foll and Goldberg, 2004; Merritt et al., 2008) and the nicotine-induced enhancement of dopamine levels in the NAc (Cohen et al., 2002).

### Chronic nicotine and withdrawal

The pathological motivational state that characterizes dependence on nicotine involves the appearance of negative affective states when nicotine exposure is discontinued (i.e., nicotine withdrawal). These may involve disruptions of the same neurobiological mechanisms that are involved in the positive reinforcing effects of the drug (i.e., within-system neuroadaptations) and recruitment of stress systems (e.g., between-system neuroadaptations). This negative affective state may represent a negative reinforcer that will enhance the incentive value of nicotine, leading to increased nicotine intake in an attempt to alleviate the negative emotional state (Solomon and Corbit, 1973; Koob and Le Moal, 2001, 2008; Koob, 2008, 2010).

Spontaneous or precipitated withdrawal from chronic nicotine produces anxiety-like behavior, CPA, and elevations of brain reward thresholds (Balerio et al., 2004; Jackson et al., 2008; Johnson et al., 2008). These affective and reward deficits likely involve downregulation of dopaminergic neurotransmission in the mesocorticolimbic system. Withdrawal from chronic nicotine results in decreased tonic firing of dopamine neurons in the VTA (Grieder et al., 2012) and decreases dopamine levels in the NAc (Fung et al., 1996; Hildebrand et al., 1998). Chronic exposure to nicotine produces a desensitization of nAChRs (Dani and Heinemann, 1996; Fenster et al., 1999; Picciotto et al., 2008) and an upregulation of nAChRs (Marks et al., 1983, 1992; Changeux et al., 1984; Dani and Heinemann, 1996; Koob and Le Moal, 2005). However, differences exist between nAChRs. For example, brain nicotine concentrations in an average smoker reach levels sufficient to desensitize α4β2 nAChRs without affecting α7 nAChRs, which requires much higher concentrations (Wooltorton et al., 2003). Glutamate release is regulated by α7 nAChRs located presynaptically (Marchi et al., 2002). Thus, during nicotine exposure, desensitization of α4β2 nAChRs on GABAergic neurons will suppress GABA release and inhibit dopamine neurons in the VTA, whereas α7 nAChRs on glutamatergic afferents will remain active and increase glutamate release on dopamine neurons in this region, facilitating dopamine secretion in the NAc (Dani, 2001; Wooltorton et al., 2003). However, nicotine withdrawal produces an opposite effect, with decreases in VTA glutamate levels and increases in VTA GABA levels (Natividad et al., 2012). Consequently, antagonism of presynaptic mGluR2/3 antagonists, known to negatively modulate glutamate release (Schoepp et al., 2003), attenuates reward deficits associated with nicotine withdrawal in rodents and alleviates the depressive-like symptoms related to nicotine abstinence in humans (Kenny et al., 2003; Liechti and Markou, 2008). Inhibition of glutamate transmission by the delivery of mGluR5 antagonists in rats and knocking out mGluR5 in mice further elevates reward thresholds during nicotine withdrawal (Harrison et al., 2002; Liechti and Markou, 2007; Stoker et al., 2012).

Endogenous opioids may play an important role in the development of nicotine dependence, reflected by the resemblance between the somatic signs induced by the cessation of nicotine exposure and those of opiate withdrawal (Malin et al., 1993; Watkins et al., 2000a) and the ability of the opioid receptor naloxone to induce somatic signs of withdrawal in heavy smokers (Sutherland et al., 1995; Krishnan-Sarin et al., 1999). Naloxone administration in rodents chronically treated with nicotine induces somatic signs of withdrawal (Malin et al., 1993; Balerio et al., 2004; Biala et al., 2005), CPA, and elevations in reward thresholds (Watkins et al., 2000a,b). MOR (Berrendero et al., 2002) and proenkephalin (Berrendero et al., 2005) knockout mice chronically exposed to nicotine show reduced somatic signs of withdrawal. Interestingly, knockout of the prodynorphin gene does not impact the somatic signs of nicotine withdrawal (Galeote et al., 2009). Moreover, nicotine withdrawal is associated with increased prodynorphin expression in the NAc (Isola et al., 2008). Thus, it can be hypothesized that during chronic nicotine exposure, there is a release of opioid peptides, which leads to downregulation of MORs and upregulation of prodynorphin-KOR systems. These opposing effects may combine to participate in the mediation of the somatic and affective aspects of nicotine withdrawal.

There is also evidence that 5-HT neurotransmission is involved in the mediation of nicotine dependence. Chronic nicotine treatment decreases the concentration of 5-HT in the hippocampus and increases the number of hippocampal 5-HT_1A_ receptors (Benwell and Balfour, 1979). This receptor upregulation may reflect reduced levels of 5-HT input from the median raphe nucleus, which is the main source of brain 5-HT and projects to various brain areas, including the hippocampus and amygdala (Benwell et al., 1990). During nicotine abstinence, decreased 5-HT, combined with upregulated 5HT_1_ receptors, may contribute to symptoms of depression and anxiety that are associated with 5-HT deficits (Coppen, 1967; Young et al., 1985; Markou et al., 1998) and nicotine withdrawal (Hughes et al., 1991). Indeed, antagonism of 5-HT receptors attenuates withdrawal-induced CPA in animals (Suzuki et al., 1997) and anxiety in withdrawn human smokers (West et al., 1991; Hilleman et al., 1992, 1994). Interestingly, a recent study suggests that acute nicotine activates 5-HT neurons in the dorsal raphe that are regionally distinct from those involved in nicotine withdrawal (Sperling and Commons, 2011).

### Stress in nicotine dependence

Convergent lines of evidence (Koob and Le Moal, 2001, 2005) suggest that stress [e.g., corticotropin-releasing factor (CRF) and orexin] and anti-stress [e.g., neuropeptide Y (NPY)] systems are involved in the emotional and motivational aspects of drug dependence (see Bruijnzeel, 2012, for an extensive review) and are largely localized to the extended amygdala, a forebrain macrostructure composed of the bed nucleus of he stria terminalis (BNST), central nucleus of the amygdala (CeA), and NAc shell (Heimer and Alheid, 1991; Smith and Aston-Jones, 2008).

#### Corticotropin-releasing factor

Nicotine self-administration increases the release of adrenocorticotropic hormone (ACTH) and cortisol/corticosterone (CORT; Donny et al., 2000; Chen et al., 2008). Evidence suggests that while CORT facilitates the reinforcing effects of drugs in non-dependent subjects, high circulating levels of CORT, as a result of repeated drug use, can feed back to shut off the hypothalamic-pituitary adrenal (HPA) axis and sensitize extrahypothalamic CRF systems, contributing to escalated and compulsive drug intake (Vendruscolo et al., 2012). CRF is a neuropeptide that has three paralogs – Ucn 1, 2, and 3 – and is involved in regulating the neuroendocrine autonomic and behavioral responses to stress (Vale et al., 1981, 1983; Dunn and Berridge, 1990; Koob, 1999). Two G-protein-coupled CRF receptors have been identified: CRF_1_ and CRF_2_. Notably, although CRF and Ucn 1 have high selectively for the CRF_1_ receptor, Ucn 2 and Ucn 3 have high selectivity for the CRF_2_ receptor (Bale and Vale, 2004). While activation of the CRF_1_ receptor leads to increases in anxiety-like behavior, activation of the CRF_2_ receptor generally triggers a compensatory anti-stress response. For example, selective CRF_1_ antagonists have been shown to reduce anxiety-like behavior in animals (Griebel et al., 1998; Deak et al., 1999; Zorrilla et al., 2002), whereas the CRF_2_ receptor agonist Ucn 3 decreases behavioral stress responses (Valdez et al., 2002, 2003). Various findings suggest that recruitment of CRF–CRF_1_ systems, particularly in regions of the extended amygdala, may be involved in producing the negative emotional states during withdrawal or protracted abstinence from chronic nicotine. First, precipitated nicotine withdrawal increases Fos expression (i.e., neuronal activation) in the CeA. Second, CRF levels in the basal forebrain (Matta et al., 2007) and CeA (George et al., 2007) are elevated during nicotine withdrawal. Third, the elevation of reward thresholds induced by nicotine withdrawal is attenuated by intracerebroventricular or intra-CeA infusion of the CRF_1_ antagonist d-Phe CRF_12–41_ and non-specific CRF antagonist α-helical CRF_9–41_ (Bruijnzeel et al., 2009; Marcinkiewcz et al., 2009; Bruijnzeel, 2012) but not a CRF_2_ antagonist (Bruijnzeel et al., 2009). Infusion of d-Phe CRF_12–41_ into the NAc shell, another region of the extended amygdala, also blocks the withdrawal-induced elevation in reward thresholds (Marcinkiewcz et al., 2009). Fourth, a CRF_1_ antagonist (MPZP) administered systemically attenuates the abstinence-induced increases in nicotine intake and nicotine withdrawal-induced anxiety-like behavior (George et al., 2007). Finally, CRF_1_ antagonists administered systemically attenuate the escalated intake of heroin and cocaine in rats with extended access to the drug (Specio et al., 2008; Greenwell et al., 2009).

#### Neuropeptide Y

Neuropeptide Y is a 36-amino-acid polypeptide with powerful anxiolytic-like properties in various animal models of anxiety and stress (Heilig and Murison, 1987; Broqua et al., 1995; Sajdyk et al., 1999; Tovote et al., 2004). The involvement of NPY in addiction was mainly studied with regard to alcohol dependence, with alcohol-preferring rats having lower basal levels of NPY in the CeA that correlate with greater levels of anxiety-like behavior compared with alcohol non-preferring rats (Suzuki et al., 2004; Pandey et al., 2005). Moreover, viral vector-induced overexpression of NPY in the CeA decreases alcohol intake in alcohol-dependent rats (Thorsell et al., 2007). These results suggest that downregulation of the NPY system in the CeA may mediate the transition from non-dependent to dependent alcohol intake. The role of NPY in nicotine dependence has been less studied. Rylkova et al. (2008) report that NPY prevents the somatic signs of withdrawal but not elevation in brain reward thresholds that result from precipitated nicotine withdrawal in rats. Yet, abstinence from nicotine induced anxiety-like behavior that was associated with a decreased ratio of NPY to CRF in the amygdala, suggesting an allostatic change in both stress and anti-stress neuropeptide systems (Slawecki et al., 2005; Aydin et al., 2011).

#### Norepinephrine

Several lines of evidence suggest that norepinephrine (NE) signaling from the nucleus tractus solitarius (NTS) to extended amygdala mediates the aversive effects of opiate and cocaine withdrawal (e.g., anxiety-like behavior; Smith and Aston-Jones, 2008). Moreover, morphine withdrawal enhances subsequent morphine-induced CPP, which is reduced by delivery of the α_2_-adrenoceptor agonist clonidine (Nader and van der Kooy, 1996). The role of NE in nicotine dependence has been less explored, but clonidine appears to decrease anxiety and irritation associated with smoking cessation and promote abstinence (Prochazka et al., 1992; Gourlay et al., 2004). The few animal studies conducted have yielded conflicting results. Deficits in brain reward function during nicotine withdrawal were attenuated by antagonism of α_1_-adrenoceptors (Bruijnzeel et al., 2010) and antagonism of α_2_-adrenoceptors in another study (Semenova and Markou, 2010). This is puzzling given the positive effect of clonidine, a α_2_ agonist, in human abstinent smokers. More studies are needed to clarify the role of NE in nicotine dependence.

#### Orexin/hypocretin

Orexin A (hypocretin-1) and orexin B (hypocretin-2) are neuropeptides that have two known receptors, Hcrt-r1 and Hcrt-r2, and regulate several processes, including arousal (Sutcliffe and de Lecea, 2002; Taheri et al., 2002) and stress responses (Baldo et al., 2003; Winsky-Sommerer et al., 2004). Orexin/hypocretin neurons are especially abundant in the lateral hypothalamus and project to various brain regions, including the extended amygdala (Peyron et al., 1998; Baldo et al., 2003). Interestingly, intracerebroventricular infusion of orexin A induces Fos activation in approximately half of the CRF-containing neurons in the CeA (Sakamoto et al., 2004). Orexin neurons also receive inputs from the amygdala (Sakurai et al., 2005), and a possible positive feedback circuit between hypothalamic orexin neurons and amygdala CRF neurons has been suggested (Corrigall, 2009). Indeed, dependent smokers during early withdrawal show a significant negative correlation between hypocretin plasma concentration and nicotine craving (von der Goltz et al., 2010). A recent study reports that nicotine withdrawal increases hypocretin cell activity in the hypothalamus and that the hypocretin-1 receptor antagonist SB334867 as well as preprohypocretin knockout attenuate somatic nicotine withdrawal signs in mice (Plaza-Zabala et al., 2012). This study also revealed that the hypothalamic paraventricular nucleus (PVN) is strongly involved in this effect. Infusion of SB334867 into this region attenuates the somatic signs of withdrawal.

#### Nociceptin/orphanin FQ

Nociceptin/orphanin FQ is a 17-amino-acid peptide that shows structural homology with the dynorphin A peptide (Reinscheid et al., 1995) and binds to the nociceptin/orphanin peptide (NOP) receptor. Nociceptin/orphanin FQ and NOP receptors are distributed throughout the central nervous system, with relatively high densities in the extended amygdala, PFC, and VTA (Neal et al., 1999). Nociceptin/orphanin FQ generally inhibits stress responses by functionally antagonizing CRF activity (Ciccocioppo et al., 2003). Chronic exposure to alcohol decreases the levels of brain nociceptin/orphanin FQ (Lindholm et al., 2002), and activation of the nociceptin/orphanin FQ system attenuates alcohol withdrawal symptoms and reverses increased anxiety-like behavior associated with ethanol dependence (Economidou et al., 2011; Aujla et al., 2013). Nociceptin/orphanin FQ might be similarly involved in nicotine dependence. NOP receptor knockout mice, unlike wildtype mice, show a significant mecamylamine-induced CPA to nicotine withdrawal (Sakoori and Murphy, 2009).

### Escalation of nicotine intake

Unlike cocaine and opiates, daily extended self-administration sessions do not induce escalation of nicotine intake but rather a reduction in intake following the first daily session and stable intake afterward (Valentine et al., 1997; Kenny and Markou, 2006; O’Dell et al., 2007; Cohen et al., 2012). However, humans typically do not have continuous access to smoking but instead alternate between periods of access (daytime) and no access (nighttime). The escalation of nicotine intake only occurs when 24–48 h of abstinence are given between extended-access (21 h) sessions (Cohen et al., 2012). It is possible that escalation does not take place when given continuous access because of nAChR desensitization (see above), which requires a period ranging from a few hours to a few days to recover (Collins et al., 1990, 1994; Girod and Role, 2001). Additionally, the escalated intake of nicotine could reflect the increased incentive value of nicotine that results from experiencing a negative affective state because of recruitment of stress systems and downregulation of anti-stress systems (Koob and Le Moal, 2001; Koob, 2010). Supporting such a hypothesis, CRF levels in the CeA are increased during precipitated withdrawal. Moreover, blocking CRF_1_ receptors systemically with MPZP attenuates both the increase in anxiety-like behavior during precipitated withdrawal and increase in nicotine intake following 72 h of abstinence (George et al., 2007). In accordance with the hypothesis that emergence of a negative emotional state is required in order to observe escalation of nicotine intake is the fact that rats with limited access to nicotine self-administration (2 h/day) escalate their nicotine intake only if they are tested under withdrawal from daily exposure to nicotine vapor that is sufficient to produce a robust withdrawal syndrome (Gilpin et al., 2013).

To further support the hypothesis that negative affective symptoms drive the escalation of nicotine self-administration, possible associations between anxiety-like behavior (among other negative affective symptoms) and the escalation of nicotine self-administration will need to be explored, and the possibility that manipulation of CRF and other stress and anti-stress systems can block the escalation of nicotine intake should be examined.

## Summary and Conclusion

Animal models of the acute effects of nicotine have provided us with ample evidence regarding the reinforcing and affective effects of nicotine and neurobiological processes that mediate them. These studies support a central role for the mesocorticolimbic dopamine system and neuronal circuits that interact with it in the acute reinforcing effects of nicotine. Studies using chronic passive delivery of nicotine via intracranial or intraperitoneal routes of administration have provided evidence that chronic nicotine dysregulates nAChRs and downregulates the same neurobiological mechanisms that are involved in the positive reinforcing effects of the drug. However, most of these studies did not examine the relationships between these neurobiological alterations and motivation to consume nicotine after dependence developed. Human smokers tend to begin smoking intermittently, especially at early ages, and quickly develop initial aversive symptoms of abstinence. Their smoking behavior escalates until daily smoking reaches a stable high level that is considered compulsive. Novel models of escalated nicotine intake will allow investigation of the mechanisms that underlie the development of compulsive nicotine intake in rats. Initial evidence suggests that recruitment of brain stress systems is a key factor in this process, but further research is needed. Novel models of nicotine exposure that utilize inhalation also provide a unique opportunity to evaluate the effects of e-cigarette use and second-hand smoking exposure on the vulnerability to dependence and relapse.

## Conflict of Interest Statement

The authors declare that the research was conducted in the absence of any commercial or financial relationships that could be construed as a potential conflict of interest.
